# Evolution of focused streams for viscoelastic flow in spiral microchannels

**DOI:** 10.1038/s41378-023-00520-4

**Published:** 2023-06-06

**Authors:** Hua Gao, Jian Zhou, Mohammad Moein Naderi, Zhangli Peng, Ian Papautsky

**Affiliations:** Department of Biomedical Engineering, University of Illinois Chicago, Chicago, IL 60607 USA

**Keywords:** Viscoelastic microfluidics, Spiral microchannel, Dean flows, Elasto-inertial focusing, Nanobiotechnology, Microfluidics

## Abstract

Particle migration dynamics in viscoelastic fluids in spiral channels have attracted interest in recent years due to potential applications in the 3D focusing and label-free sorting of particles and cells. Despite a number of recent studies, the underlying mechanism of Dean-coupled elasto-inertial migration in spiral microchannels is not fully understood. In this work, for the first time, we experimentally demonstrate the evolution of particle focusing behavior along a channel downstream length at a high blockage ratio. We found that flow rate, device curvature, and medium viscosity play important roles in particle lateral migration. Our results illustrate the full focusing pattern along the downstream channel length, with side-view imaging yielding observations on the vertical migration of focused streams. Ultimately, we anticipate that these results will offer a useful guide for elasto-inertial microfluidics device design to improve the efficiency of 3D focusing in cell sorting and cytometry applications.

## Introduction

The tremendous clinical potential of circulating tumor cells (CTCs)^1–3^ and circulating extracellular vesicles (e.g., exosomes)^4^ for liquid biopsy in cancer diagnostics and precision medicine has been driving the burgeoning development of microfluidic devices for cell sorting and isolation. The laminar flow nature of these devices permits the manipulation of fluids and suspended cells or particles with remarkable spatial and temporal precision. Both biophysical and biochemical properties of the cells and particles are widely exploited in these devices as markers for generating differentiated spatial positioning inside the devices by adding either external^5^ or internal differentiating fields^6,7^. A popular approach to cell sorting in microfluidic channels relies on the inertia of the surrounding fluid, with inertial effects driving cells across flow streamlines into equilibrium positions. In straight microfluidic channels with square cross-sections, cells focus in four equilibrium positions at the center of each side wall^8^. In channels with rectangular cross-sections, the number of equilibrium positions is reduced to two near the centers of the larger side walls^9^. Introducing channel curvature disrupts this equilibrium balance due to the emergence of secondary flows that form counter-rotating Dean vortices and create two vertical equilibrium positions near the inner convex side wall^10,11^.

The majority of the previous work on cell sorting has been performed in Newtonian flows. However, biofluids such as blood are non-Newtonian^12^, which can impact device performance and cell separation effectiveness. These biofluids are generally viscoelastic in nature, making cell sorting challenging. Fortunately, fluid viscoelasticity can offer unique opportunities to focus cells into different cross-sectional locations in a microfluidic channel, depending on their size^13–15^. Recent studies have shown that the viscoelastic focusing of particles is preferable when forming 3D single stream focusing^13,16^ and in the enrichment of submicrometer particles^4,17,18^. This distinct particle focusing behavior in viscoelastic fluids is attributed to their unique rheological properties. These fluids consist of both viscous and elastic components and thus behave like viscous fluids in some circumstances and as elastic fluids in others. In elasticity-dominated flows with negligible inertia, particles migrate into lower shear regions, forming a single stream in the center of a circular straight microchannel^19^. In square straight channels, due to the radially asymmetric shear distribution, particles focus at the channel center and the four corners^13^, while in rectangular channels, particles become confined into a broad band near the central plane of the channel^20^. When fluid inertia is not negligible (i.e., elasto-inertial flows), multiple equilibrium positions can be reduced to a single 3D position by regulating the synergetic combination of the elasticity and inertial effects on particles^13^. In rectangular straight channels, Seo et al.^21^ found that particles concentrated and aligned near the centerline of the channel due to the asymmetric normal stress distribution at the channel walls. Li et al.^22^ demonstrated that at low flow rates, the elastic lift force dominated particle focusing behavior, while at high flow rates, the inertia lift force became dominant.

Although a number of recent publications^2,23,24^ have reported on particle and cell focusing in elasto-inertial flows, their attention has been on separation applications in straight microchannels rather than on the underlying principles in spiral microchannels. In part, this is due to challenges associated with investigating particle migration in 3D spiral channels. The three major challenges include (1) the variation of force balance in the downstream direction due to the planar spiral geometry, (2) the complex interactions among inertial lift, elastic, and Dean drag forces, and (3) the lack of direct observation from the side view^25^. Xiang et al.^26^ attempted to explain the observed migration behavior using a complex six-step model. In a more recent work, Feng et al.^27^ demonstrated that the focusing positions of particles were dependent on synergetic hydrodynamic forces. However, the evolution of particle migration along the channel downstream length was not described, leading to an incomplete model and prediction of the particle focusing position. Lee et al.^28^ investigated particle lateral position dynamics along the channel downstream but in a single spiral channel and narrow flow rate range (0.83 to 12.5 μL/min). Kumar et al.^29^ and others^14^ showed the hydrodynamic force balance in the cross-section, with particles reaching stable 3D focusing at high *Re*. However, the lack of a direct observation of particle vertical position in the flow puts in question the accuracy of the inferred viscoelastic migration mechanism in 3D spiral channels. Thus, despite these efforts, a clear understanding of the 3D migration of particles in spiral channels in elasto-inertial flows is still lacking.

In this work, we aim to improve the understanding of the underlying mechanisms of particle elasto-inertial migration in spiral channels. We systematically investigate the evolution of neutrally buoyant particles along the downstream length from top and side views in spiral microchannels and explore the effects of device geometry, flow direction, and fluid elasticity on particle focusing dynamics over a wide range of flow rates. Our results illustrate the full focusing pattern along the downstream channel length under these diverse conditions. In addition, sideview imaging yields observations on the vertical migration of focused streams. We hope that these results will offer a useful guide for elasto-inertial microfluidic device design to improve the efficiency of 3D focusing in cell sorting and cytometry applications.

## Materials and methods

### Design and fabrication of microfluidic devices

Four Archimedean spiral devices (R1, R2, R4 and R6) were used in this work, with 1 mm, 2 mm, 4 mm and 6 mm initial radii of curvature. Channel cross-sectional dimensions were fixed at 250 µm in width and 50 µm in height, corresponding to an aspect ratio AR = 0.2. The spacing between channels was set at 250 µm. A single outlet was located at the center of each spiral, while a single inlet with a debris filter was located at the circumference. The details of the channel layout and dimensions are summarized in Fig. S1.

Channels were fabricated in polydimethylsiloxane (PDMS) using the standard soft lithography process with dry photoresist masters, as we detailed previously^30^. Briefly, 3 silicon wafers were dehydrated for 15 min on a 225 °C hotplate, laminated with a 50 μm thick film (ADEX 50, DJ Microlaminates Inc., Boston, MA, USA) and baked for 5 min on a 65 °C hotplate. Next, the wafers were exposed to UV light (I-Line 365 nm, Optical Associates Inc., USA) for 33 s at 10 mW/cm^2^ through a mask plate in hard contact. The wafers were developed in cyclohexanone (98%, Acros Organics, USA), washed with IPA and DI water, air dried, and baked for 90 min on a 170 °C hotplate (Thermo Fisher Scientific, Inc., USA). PDMS (Sylgard 184, Dow Corning, Midland, MI, USA) was mixed with curing agent in a 10:1 ratio, cast on the fabricated master, degassed for 90 min in a vacuum oven, and cured on a hotplate at 60 °C for 4 h. PDMS replicas were peeled off, and ports were cored using a 1.6 mm diameter biopsy punch (Miltex, Japan). Devices were bonded to standard microscope glass slides using oxygen plasma treatment at 10 W for 20 s (PE-50, Plasma Etch, Inc., Carson City, NV, USA), baked for 60 min on a hot plate at 80 °C, and allowed to cool to room temperature before use. For side-view imaging, devices were mounted vertically; to do this, the edges of PDMS replicas were cut using a sharp blade orthogonal to the spiral pattern, then placed vertically on uncured PDMS in a Petri dish (Fisher Scientific Inc., MA, USA) and cured on a hotplate at 60 °C for 4 h to improve optical transparency and resurface the cutting edge.

### Sample preparation

Polyethylene oxide (PEO) was used as the viscoelastic fluid in this work. Six concentrations were prepared (50 ppm, 250 ppm, 500 ppm, 1000 ppm, 2500 ppm and 5000 ppm) by mixing PEO powder with a molecular weight of 2,000,000 Da (Sigma Aldrich., USA) into 22% (w/v) glycerin (Fisher Scientific, Inc., USA) and deionized water (DI). A 5% (w) NaCl solution was added to match the density of polystyrene particles (1.05 g/cm^3^). Solutions were gently mixed on a stirring plate (Thermo Fisher Scientific, Inc., USA) at room temperature for 24 h. The rheological properties of the prepared solutions are summarized in Table 1.

**Table 1:** Rheological characteristics of the 5 wt% water-based NaCl Newtonian fluid and 22 wt% glycerol/DI water-based non-Newtonian and Newtonian fluids used in this study^13,28,54–56^

Rheological properties	Water solutions (wt%)		PEO solutions (ppm)					
	NaCl (5%)	Glycerol (22%)	50	250	500	1000	2500	5000
Density, ρ (g/cm^3^)	1.05	1.05	1.05	1.05	1.05	1.05	1.05	1.05
Zero-shear viscosity, η (mPa ∙ s)	1	2.05	2.08	2.46	3.12	4	16	32
Overlap concentration, c* (ppm)			858	858	858	858	858	858
Concentration ratio, c/c*			0.06	0.29	0.58	1.17	2.91	5.83
Effective relaxation time, λ_e_ (ms)			2	5.8	9.1	12.4	23	40

To investigate the focusing behavior, solutions of fluorescent 25 μm-diameter polystyrene beads (Polysciences Inc., USA) were prepared with a final volume fraction of 0.03% (v/v). Tween 80 (Fisher Scientific, USA) was added at 0.1% (v/v) to minimize aggregation and avoid channel clogging. Larger 25 μm-diameter beads (blockage ratio *β* = 0.3 since *D*_*h*_ = 83.3 μm) were used to observe the complete evolution of the focused particle streams in a relatively short downstream distance due to the high viscosity of the PEO solutions that limited the channel length. The stronger fluorescent signal of these larger particles enables capture of the focusing trajectories in flows with a lower particle concentration to avoid particle‒particle interactions.

### Experimental setup and flow imaging

Particle suspensions were loaded into a 10 mL syringe with a Luer lock interface and connected to microfluidic devices using 0.06” PTFE tubing (Cole-Parmer). A programmable syringe pump (Legato 201, Kd Scientific, USA) was used to drive particles into devices with preset flow rates on a vibration isolation table. The flow rate was varied from 50 μL/min to 350 μL/min. Fluorescence imaging was accomplished using an inverted microscope (IX83 Olympus, Inc., USA) with a 16-bit sCMOS camera (Zyla 5.5, Andor Technology Ltd, Belfast, UK). Fluorescence images of the top view were taken at quarter-circle positions throughout the channel length with an exposure time of 150 μs to acquire particle flow trajectories by using a 20× objective with a high numerical aperture (NA = 0.7). CellSense software (Olympus, Inc.) was used to sequentially obtain 150 images at each position. Brightfield images of the top view were captured with a high-speed camera (AX 200 Mini, Photron USA, Inc.) at a frame rate of up to 10,000 fps depending on the flow rate, with an exposure time of 1 μs for obtaining the particle distribution probability and height position along the channel height.

The sideview images were obtained in spiral channels vertically placed on the microscope stage using a high-speed camera (AX 200 Mini, Photron USA, Inc.) using the approach reported in previous studies^10,31^. Brightfield side view images of the particles were taken with a 10× objective with a long (10 mm) working distance using the camera settings described above.

At least 150 images were stacked and used to measure the distance between the closest wall and center of each particle. At least 200 measurements were made for each flow rate. Using the distance measurements, a kernel density estimate (KDE) plot was used to generate the probability distribution function (PDF)^32–34^ in the direction of channel height (RStudio, Inc). This calculation was performed for each flow rate, with a total of 1617 particles measured.

### Data analysis

The particle migration trajectories were created by stacking consecutive frames, and the particle lateral and downstream positions from the top view and side view were manually measured using ImageJ and NIH software. The trajectories of particle migration along the downstream were created by consecutively splicing the stacked fluorescence streak images at each observation position. Three fluorescence intensity profiles were measured per imaging position by using ImageJ and then averaged in a custom Python program. Streamline width was defined as the fluorescence intensity FWHM (full width at half maximum) across the channel width at each position where the intensity profile was above the 10% threshold to avoid channel edge reflection interference. Focusing was defined as the streamline FWHM being smaller than 2× the particle diameter. The particle probability density along the channel height is an empirical function plotted by the kernel estimator in R software (RStudio Team) from hundreds of single particle vertical positions. OriginPro (OriginLab Corporation, USA) was used to analyze the data and plot the results.

### Numerical model

COMSOL Multiphysics 5.6® was used to model the evolution of flow variables. Only the steady-state flow fields without particles were modeled. To overcome convergence issues due to a high Wi number (Wi > 9) at flow rates Q > 100 µL/min, only a segment of the spiral channel was modeled with appropriate periodic boundary conditions. Due to the symmetric nature of the flow with respect to the z-axis, the simulations were performed only for the lower half of the channel using the symmetry boundary condition to reduce computational cost. Stationary laminar flow equations [Eqs. 1–2] were solved with the Giesekus model as the constitutive equation^35^ [Eqs. 3–4] to capture the viscoelastic and shear-thinning behaviors of the PEO solution^13,36,37^:

1 $$\rho ( {{{{\mathrm{u}}}} \cdot \nabla } ){{{\mathrm{u}}}} = \nabla \cdot ( { - {{{\mathrm{p}}}}I + \mu _s({{{\mathrm{L}}}} + {{{\mathrm{L}}}}^T) + {{{\mathrm{T}}}}_{{{\mathrm{e}}}}} )$$

2 $$\nabla \cdot {{{\mathrm{u}}}} = 0$$

3 $$\lambda {{{\dot{\mathrm T}}}}_{{{\mathrm{e}}}} + \left( {1 + \frac{{\alpha \lambda }}{{\mu _p}}{{{\mathrm{T}}}}_{{{\mathrm{e}}}}} \right){{{\mathrm{T}}}}_{{{\mathrm{e}}}} = \mu _p\left( {{{{\mathrm{L}}}} + {{{\mathrm{L}}}}^{{{\mathrm{T}}}}} \right)$$

4 $${{{\dot{\mathrm T}}}}_{{{\mathrm{e}}}} = \left( {u \cdot \nabla } \right){{{\mathrm{T}}}}_{{{\mathrm{e}}}} - {{{\mathrm{L}}}} \cdot {{{\mathrm{T}}}}_{{{\mathrm{e}}}} - {{{\mathrm{T}}}}_{{{\mathrm{e}}}} \cdot {{{\mathrm{L}}}}^{{{\mathrm{T}}}}$$

Here, *u* is the velocity vector; *L* denotes the velocity gradient tensor; *ρ*, *μ*_*s*_, and *I* are the fluid density, solvent viscosity, and identity matrix; *T*_*e*_ is the extra elastic stress tensor; and ${{{\dot{\mathrm T}}}}_{{{\mathrm{e}}}}$ is the upper-convective time derivative. In the Giesekus model, *λ* and *μ*_*p*_ are the relaxation time and the viscosity of the polymer part of the fluid, respectively, and *α* is the mobility factor related to shear thinning^38^.

A global ODE equation was added to the model to generate the desired flowrate by adjusting the pressure difference between the segment inlet and outlet. A tetrahedral mesh with 126,000 elements was used to discretize the domain, and the spiral segment length was fixed at *π*/20 radians. The mesh configuration details are given in Fig. S2. The dependency of the simulation results on the number of mesh elements and the spiral segment length were assessed by plotting the radial velocity along half the channel height in a convergence test; these results are provided in Fig. S3.

### Validation of the numerical model

The numerical model is based on the Giesekus fluid to capture the viscoelastic and shear-thinning properties. However, due to the lack of systematic numerical results in the existing literature, it is not possible to quantitatively validate the flow variables (*De* flow, first and second normal stress difference, etc.). Thus, we validated the correctness of our numerical results qualitatively. Based on our simulation results, the center of maximum velocity in the downstream direction shifted toward the inner wall, which is consistent with Feng et al.^27^. Furthermore, the vortices formed in the viscoelastic flow were clearly the superposition of the two counter-rotating vortices due to the channel curvature, and the four corner vortices formed due to the nonzero second normal stress difference, as an established viscoelastic phenomenon in a Giesekus fluid^39^. In pure inertial flows, however, the secondary flow only has the effect of the two counter-rotating vortices due to channel curvature only, as reported by us and others^40–42^. Because of the additional complexity introduced to the model and numerical challenges of simulating flows with high *Wi* numbers^43^, we were not able to achieve convergence for our model when adding the particle to the domain.

## Results and discussion

### Operation of spiral devices

Particle migration in spiral channels has been reviewed by us^17,25^ and others^41,44^. In viscoelastic fluids, this is dependent on the interaction of three hydrodynamic forces: inertial lift force, curvature-induced Dean drag force, and elastic force. It is now well accepted that inertial focusing occurs when the particle Reynolds number *Re*_*p*_ ≥ 1^45^ (*Re*_*p*_ = *Re(a/D*_*h*_*)*^*2*^ = *ρU*_*f*_*a*^*2*^*/μD*_*h*,_ where *Re* is the channel Reynolds number, *U*_*f*_ is the average fluid flow velocity, *a* is the particle diameter, *ρ* is the fluid density, *µ* is the fluid viscosity, and *D*_*h*_ is the hydraulic diameter of the channel). As particles flow downstream, they experience an inertial lift force *F*_*s*_ induced by fluid shear, as well as a wall-induced lift force *F*_*w*_ generated by the interaction of particles and channel walls. These forces scale strongly with particle diameter and the location in the channel, with the total net lift force *F*_*L*_ acting on particles as *F*_*L*_ ∝ *ρU*_*f*_^*2*^*a*^*2*^*/D*_*h*_^*2*^ near the channel center and as *F*_*L*_ ∝ *ρU*_*f*_^*2*^*a*^*6*^*/D*_*h*_^*4*^ near the channel wall^45^. In addition to inertial lift, particles in viscoelastic fluid are subject to the elastic force, which is the strongest at channel walls and the weakest at the centerline and corners^46^. The interaction between elastic and inertial forces results in an equilibrium position near the channel centerline^47^. The nondimensional Weissenberg number (*Wi*) is used to describe the viscoelasticity of the fluid and is given as *Wi* = *λ*$\dot \gamma$, where $\dot \gamma$ is the flow shear rate and λ is the fluid relaxation time. The elasticity number (*El*) indicates the relative importance of the elastic and inertial forces in shear flows^31^ and is given as *El* = *Wi*/*Re*. When *El* >> 1, the fluid elastic force dominates, while inertial stress is dominant when *El* << 1. For Newtonian fluids, λ = 0, and thus *Wi* = 0 and consequently *El* = 0, indicating the dominance of inertial effects.

The radial centrifugal acceleration of fluid in spiral microchannels leads to the formation of two counter-rotating vortices, with the magnitude of flow described by the nondimensional Dean number (*De*) as $De = Re\sqrt {\frac{{D_h}}{{2R}}}$, where *R* is the radius of curvature. Particles entrained in these vortices are subject to Dean drag force *F*_*D*_, and near the top and bottom walls in low aspect ratio channels, the inertial lift forces are orthogonal and thus do not disrupt particle migration within vortices. In Newtonian fluids, near the outer wall, both *F*_*D*_ and *F*_*L*_ are in the same direction, and thus, particles follow the Dean vortices. In this case, *F*_*D*_ = 3πμ*a*$\bar U$_Dean_ = 5.4 × 10^−4^ πμ*aDe*^1.63^ (where $\bar U$_Dean_ is the average Dean velocity $\bar U$_Dean_ = 1.8 × 10^−4^ *De*^1.63^)^7,48–50^. Near the inner wall, however, in Newtonian fluids, the inertial and Dean forces act in opposite directions, leading to a possible force balance for particle focusing. In viscoelastic fluids, the elastic force is orthogonal to the channel walls, and thus, near the top and bottom walls, particles are also entrained in Dean vortices. Near the inner wall, however, the elastic force and Dean drag overcome the inertial lift force, and thus particles continue to follow the vortex flow. At the outer wall, the Dean drag is now counteracted by the elastic force instead of the shear-induced lift force, leading to a possible force balance for particle focusing near the center of the outer wall^25,26^.

### Effects of flow rate

**Fig. 1:**
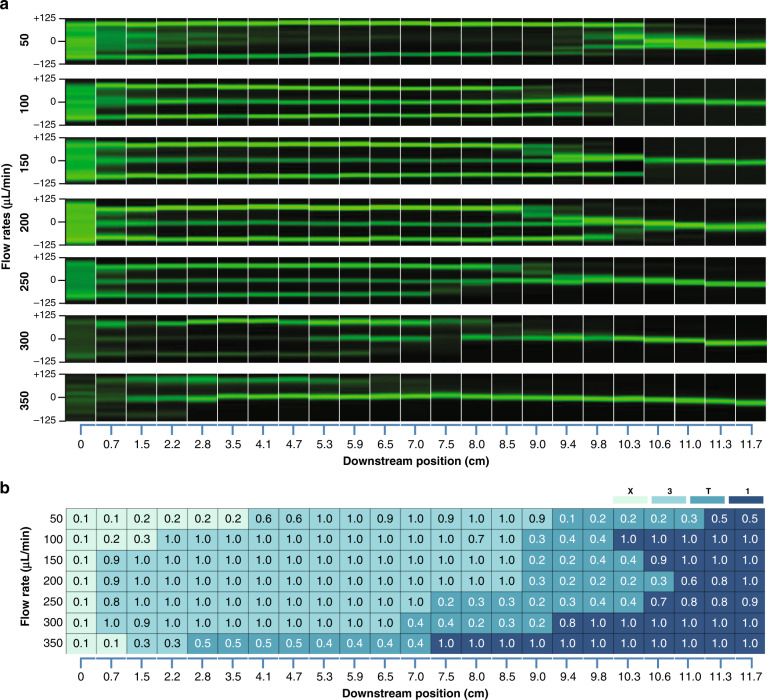
Downstream evolution of particle focusing. **a** Stacked fluorescent streak images illustrating the downstream evolution of particle focusing in the R2 spiral channel (aspect ratio AR = 0.2) at a 50–350 μL/min flow rate. The +/−125 scale indicates the normalized width of the 250 μm wide channel. The downstream position is indicated at the bottom of the panel set. **b** Heatmap illustrating the lateral migration of particles in the R2 channel. Each cell reports the focusing quality (FQ = a/FWHM) of fluorescent streams. The color of each cell indicates the unfocused (X), three-stream (3), transition (T) or single-stream (1) regions

Particle migration in spiral microchannels in a viscoelastic fluid yields a single focusing stream at the outlet. We recorded fluorescent streak velocimetry images over a wide range of flow rates from 50 to 350 µL/min in the R2 spiral channel in 500 ppm PEO solution. Spherical particles with a diameter of 25 μm were selected to observe the focusing evolution in the spiral channel. With a larger diameter, the 25 μm particles afford a higher blockage ratio (β = 0.3 since *D*_*h*_ = 83.3 μm), which permits us to visualize the full range of stream evolution in a shorter downstream length. This is an important consideration due to the high viscosity of the viscoelastic fluid, which leads to a large pressure drop. Second, the stronger fluorescent signal of these larger particles enables capture of the focusing trajectories in flow with a lower particle concentration to avoid particle‒particle interactions. The top-view fluorescent images in Fig. 1a illustrate that randomly distributed particles at the inlet develop into a single stream at the outlet. Since the 500 ppm PEO solution is viscoelastic, the outlet focused stream is near the channel centerline. Another key observation is that the randomly distributed particles at the inlet evolve into 3 streams within the first loop for all flow rates, although the transition occurs earlier at higher flow rates (and thus higher *De* and *Re*). Before the 3 streams transition into 1 at the outlet, they undergo a transition region where streams appear to merge and disappear.

While fluorescent images aid in visualizing particle trajectories at different flow rates, we developed a heatmap to quantitatively investigate focusing quality and stream evolution. The focusing quality (FQ) is the ratio of the particle diameter to the full width at half maximum (FWHM) of the stream intensity, and it approaches zero when particles are dispersed throughout the channel cross-section and reaches unity for perfect focusing. The heatmap in Fig. 1b allows us to visualize the evolution of particle streams from a random distribution (X) at the inlet to 3 streams and thorough a transition region (T) to a single stream, all of which appear to depend on the flow rate. Specifically, the initial transition to 3 streams is inversely proportional to the flow rate, taking 5.3 cm downstream at 50 µL/min but only 0.7 cm at 350 µL/min. This emergence of 3 distinct streamlines has been reported previously in low-aspect-ratio straight^51^ and spiral^26^ channels. Further downstream, the onset of the transition to a single stream appears to maintain the inverse relationship with flow rate. The transition to a single stream takes place at 11.3 cm downstream at 50 µL/min but occurs much earlier at 7.5 cm at 350 µL/min. Interestingly, although evolution to the transition region (T) occurs later at low flow rates, the length of the transition region appears to be much shorter than at higher flow rates.

### Effects of flow direction

The Dean number is inversely proportional to the square root of the radius of curvature. In a planar spiral channel, the *De* of the inner loop is higher than the *De* of the outer loop. Consequently, particles that enter the channel at the center inlet will experience a slowly decreasing Dean number. Conversely, particles that enter the channel from the outside loop will experience a slowly increasing Dean number. Since the Dean drag force magnitude is dependent on the Dean number^48^ as *F*_*D*_ ~ *De*^*1.63*^, the balance of inertial, viscous, and Dean forces that determines the particle stream lateral position and focusing quality changes with the downstream position. Most of the inertial spiral devices reported in the literature are used for cell or particle sorting flow samples from inside out, often due to device designs that must accommodate large multifaceted outputs. However, devices that do not require large output structures and are used primarily for cell or particle focusing can flow samples in either direction. Indeed, the outside-in flow direction may be advantageous, as gradually increasing the Dean number may improve the focusing quality and thus device performance.

**Fig. 2:**
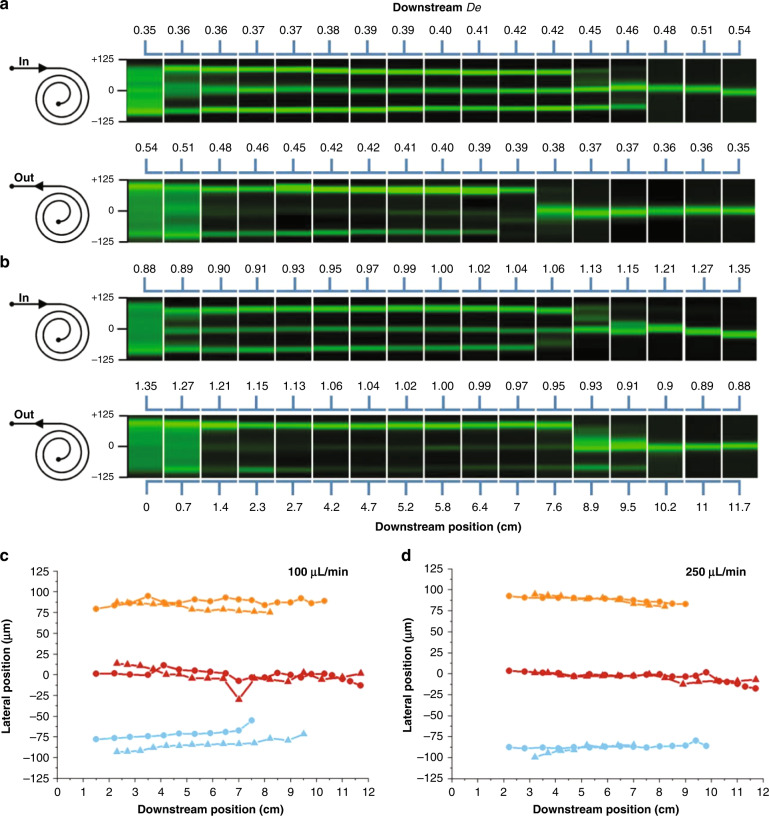
Flow direction effects on particle lateral migration. Fluorescence streak images illustrating the downstream evolution of particle focusing for opposite flow directions in the R2 spiral channel at (**a**) 100 μL/min and (**b**) 250 μL/min flows. The downstream position label is at the bottom, while the corresponding Dean number (*De*) is at the top of each panel set. Device icons on the left indicate the flow direction. Lateral position of particle streams evolving downstream of the microchannel at (**c**) 100 μL/min and (**d**) 250 μL/min flows. Blue is the inner stream, red is the central stream, and yellow is the outer stream. Circles represent data for the inside-out flow direction, while triangles represent data for the outside-in flow direction

To investigate the impact of the flow direction, we flowed particles in inside-out and outside-in directions (inlet and outlet reversed) in an R2 spiral device at 100 μL/min (low) and 250 μL/min (high) flow rates. Comparing the evolution of particle streams in both channel directions at the two flow rates presented in Fig. 2a–b, a number of observations emerge. First, for the increasing Dean number flow (outside-in), the lateral position of the single particle stream near the end of the channel gradually shifts toward the inner wall at both flow rates, whereas the lateral position of the single particle stream slightly shifts toward the outer wall for the decreasing Dean number flow (inside-out). Second, in the transition region of the increasing Dean number flow (outside-in), the outer stream is the first to migrate toward the center at a low 100 μL/min flow rate. Increasing the flow rate to 250 μL/min causes the reverse—the inner stream is the first to migrate to the center, with the outer stream beginning its migration nearly 2 cm downstream. For the decreasing Dean number flow (inside-out), the reverse occurs at each flowrate. Specifically, at 100 μL/min, the inner stream migrates toward the center first, while the outer stream is the first to migrate at 250 μL/min.

To better compare the impact of flow direction, we plotted the position of the streams in the downstream direction at 100 μL/min (low) and 250 μL/min (high) flow rates for both increasing and decreasing Dean number flows (Fig. 2c, d). The data were plotted as circles for the inside-out flow direction and as triangles for the outside-in flow direction. The inner, central and outer streams are represented with blue, red and yellow lines, respectively. These data illustrate that at both flow rates and regardless of flow direction, the randomly distributed particles at the inlet form three streams that subsequently merge into a single central stream at the outlet. At a low flow rate, the streams near the channel sidewalls appear at approximately 100 μm away from the centerline, with both merging into the central stream by 10 cm downstream. Increasing the flow rate causes these side streams to shift closer to the center, to approximately 75 μm away from the centerline, with both again merging into the central stream by 10 cm downstream. Thus, we conclude that reversing the direction of flow in these spiral microfluidic devices yields the same overall focusing behavior at the outlet.

### Particle migration along the channel height

**Fig. 3:**
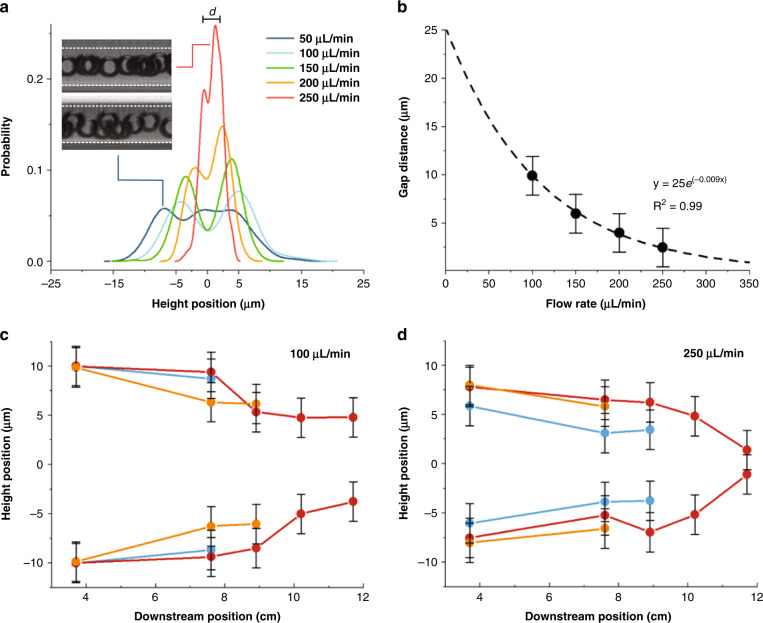
Vertical migration of particles. **a** Vertical position probability in the R2 channel for inside-out particle flow at 50–250 μL/min. The particle vertical position is measured from the particle center to the bottom of the channel. Each plot corresponds to the particle vertical position probability based on *n* = 455, *n* = 301, *n* = 302, *n* = 268, and *n* = 291 individual measurements. Inset illustrates representative brightfield images obtained using side-view imaging at 50 and 250 μL/min. **b** Gap distance (*d*) between particle streams as a function of flow rate. Measurements of particle vertical positions as a function of downstream position at flow rates of (**c**) 100 μL/min and (**d**) 250 μL/min. The vertical positions were measured from particle probability peaks from 100 individual measurements along the channel downstream length. The red, orange, and blue lines represent the particle position in the focal planes near the center, outer wall, and inner wall, respectively. Due to channel deformation, empirical ±2 μm systematic error bars were added in (**b**), (**c**), and (**d**)

We next investigated the migration of particles along the channel height near the outlet. It has been suggested by Toner^52^ and others^31^ that two focusing positions near each other are present at the outlet in inertial flows. In viscoelastic flows, however, this remains unclear due to the lack of direct observations. Additionally, side-view imaging can aid in explaining the observed 3-T-1 focusing behavior. Thus, we mounted a spiral microchannel vertically to visualize particle focusing along the channel side walls at the outlet. A similar approach was used by us^10^ and others^31^ in the past to visualize particle focusing in the side view. Due to the bonding strength of the vertical mount, we were limited to flow rates below 250 μL/min. Next, we investigated particle focusing behavior in 500 ppm PEO (*El* = 2.6). The particle probability density (PPD) obtained from brightfield side-view images, which is analogous to the line scan in fluorescent images, is presented in Fig. 3a for the R2 channel at 50–250 μL/min inside-out flows. At a lower flow rate, the particles appear as a board and poorly focused ~30 μm wide stream. The stacked brightfield image inset at 50 μL/min confirms this. At a flow rate >100 μL/min, the two distinct streams migrate closer to the centerline, with the gap distance between the streams decreasing from ~10 μm at 100 μL/min to ~2.5 μm at 250 μL/min. The stacked brightfield image inset at 250 μL/min confirms a stream at the centerline. Collectively, these results confirm the presence of two vertical streams in viscoelastic flow for each top-view stream and differ from the existing literature that assumes a single stream in the vertical center plane. This is a key new finding that helps explain the evolution of the focusing streams.

Plotting the gap distance between the vertical peaks reveals that streams shift exponentially closer to the centerline with increasing flow rate at the outlet of the 11.7 cm long channel (Fig. 3b). The correlation equation for gap distance is *d (μm)* *=* 25 *e*^*-0.009 Q*,^ where *Q* (μL/min) is the flow rate (R^2^ = 0.999). The expression suggests that at <50 μL/min (*Re* < 2) flow rates, the gap distance would be >15 μm, with particles located less than 5 μm from the top and bottom walls. At a low flow rate, the inertial lift *F*_*L*_ is weak, and thus, the particles are not vertically focused to form gaps, which matches the experimental data in Fig. 3a that shows particles being unfocused at the lowest flow rate. Interestingly, these data also suggest that at >350 μL/min (*Re* > 15) flow rate, the gap distance will decrease to *d* < 0.5 μm and thus essentially yield single stream focusing. At a flow rate of 250 μL/min, the gap was reduced to 2.5 μm in the z-direction, yielding a 3D focusing quality of 91% and thus essentially a single stream.

To explore the downstream evolution of particle migration, we measured the vertical positions of the two particle streams along the channel length. The data in Fig. 3c, d illustrate that particle streams progressively migrate toward the channel centerline in the downstream direction for all three streams observed from top view. Interestingly, the results show different toward-center migration speeds at low and high flow rates for the streams near the inner wall and outer wall, which coincides with the opposing disappearing order of the side streams observed in the top view (Fig. 2). At 100 µL/min, the side streams near the outer wall migrate faster toward the center vertically than those near the inner wall. Note that near the vertical center plane, the Dean drag is toward the outer wall, which prevents the disappearance of the outer streams and leads to the later merging of the outer streams. On the other hand, at higher flow rates, these side streams near the outer wall are closer to the top and bottom walls, where the Dean drag is toward the center, which leads to the fast merging of these streams to the center streams observed from the top view. Additionally, these results indicate that the two particle streams migrate toward the centerline at a faster rate at higher flow. The results also suggest that longer spiral channels will yield vertical streams closer to each other. For example, for the two particle streams to migrate to only 1 μm separation, at 100 μL/min, a full 25 cm downstream flow will be needed, but at 250 μL/min, only 17 cm is necessary. We next investigated the effects of channel curvature and Dean force on particle lateral migration.

### Effects of curvature

**Fig. 4:**
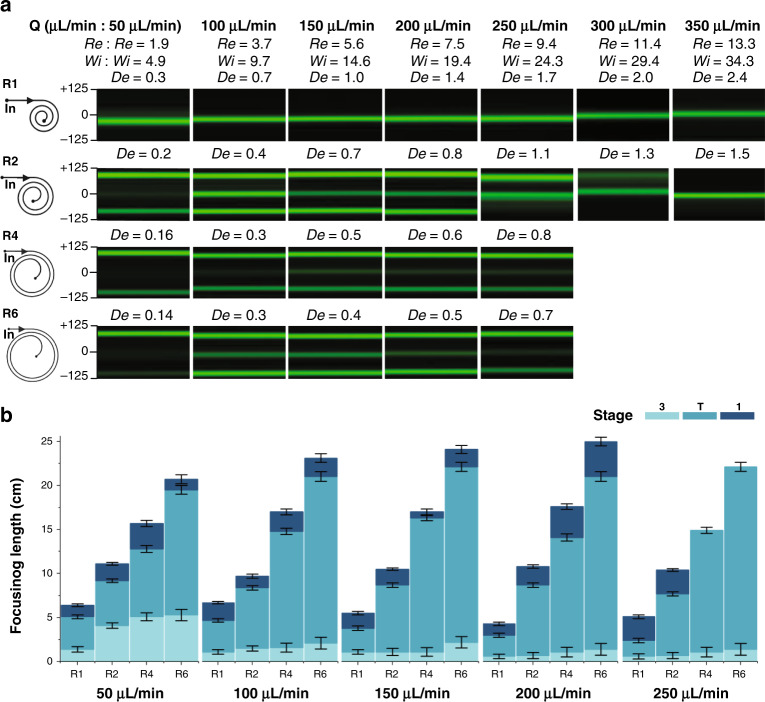
Curvature effects on particle focusing. **a** Fluorescent images demonstrate particle trajectories in four devices (R1, R2, R4, and R6) at 8 cm downstream in 500 ppm PEO at 50–350 μL/min. The flow parameters are at the top of each column. The corresponding *De*, which changes with curvature, is at the top of the fluorescent images. **b** Bar plots illustrating the downstream migration distance to evolve three or one stream for 50–250 μL/min. Error bars indicate ¼ loop length from the observed position

Fluorescence streak velocimetry illustrates that curvature, as expected, significantly impacts particle migration in spiral channels (Fig. 4a). We tested devices with radii of curvature ranging from 1 mm (R1) to 6 mm (R6) with the same cross-section (250 µm wide × 50 µm height) at 50~350 µL/min in 500 ppm PEO (*El* ~ 2.6). To decouple the downstream and curvature effects on particle lateral migration, the channel length was fixed at 8 cm. We found that in larger curvature devices (R4 and R6), the smaller effects of *F*_*D*_ particles are confined in three distinct streams near the channel sidewalls and centerline for flow rates of 50 to 250 μL/min. In smaller curvature devices (R1 and R2), with stronger *F*_*D*_ effects, particle streams reduce to a single stream near the centerline. However, in the larger curvature devices (R4 and R6), where migration is at the same *Re* but at smaller *De*, particles persist in three streams. This suggests that the persistent three streams result from inertial–elastic interactions. These results agree with observations reported by Xiang et al.^26^. In the R1 device, particle equilibrium progressively shifts from the inner wall to the outer wall with increasing flow rate. This suggests that the curvature-induced Dean effect contributed to particle lateral migration, yielding particle streams near the sidewalls dismissed and refocused near the centerline of the channel with increasing *De* (smaller radius of curvature).

Examining the focusing length at 50 μL/min to 250 μL/min for each device curvature (Fig. 4b) reveals that smaller curvature devices require shorter downstream lengths to produce a single stream. This suggests that particles rapidly move into three streams from random distribution at the inlet, indicating fast lateral migration velocity occurring at first. At a fixed length of the spiral channel, the smaller innermost curvature provides stronger Dean effects, and the multiple stream positions decrease to one. At 250 μL/min, we found that at the same flow rate and downstream length with increasing *De*, particles initially migrated into three streamlines, transitionally appeared as two streams near the centerline in R2, and finally migrated into a single stream near the channel centerline in device R1. This matches the results discussed above, where increasing *De* leads to a reduction of multiple streamlines to one and an improvement in focusing quality.

To explore the evolution in a larger curvature device, we extended the downstream length from inlets to outlets and plotted the particle trajectory heatmap of devices R1, R4 and R6 in Fig. S4. At high flow rates, particles begin migrating to their final equilibrium position near the centerline in R4 and R6. This observation was similar to the R2 device at low flow rates. As discussed above, the particle rapidly migrated from the channel bulk toward the centerline under the influence of elastic force and inertial lift force. The dominant elastic force along the channel height pre-focused and aligned particles near the centerline. On the other hand, because of the velocity profile, the shear gradient lift force pushed the particles away from the center toward the channel walls, and the particles were finally focused at both side walls. This confirmed that the particle equilibrium position strongly depended on device curvature and that Dean effects played an important role in particle migration, which is in agreement with earlier observations in Fig. 1.

### Effects of elasticity

**Fig. 5:**
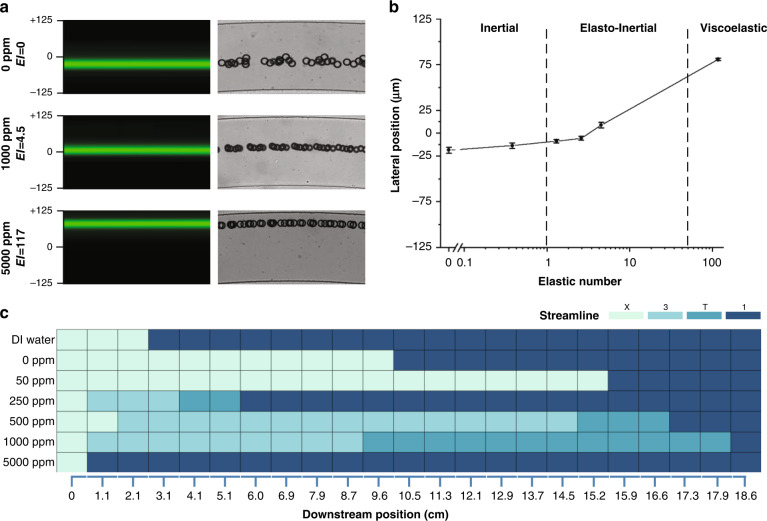
Elasticity effects on particle focusing. **a** Fluorescent and brightfield images of particle focusing in 0 ppm, 500 ppm, and 5000 ppm PEO solutions observed at the outlet. **b** Single-stream focusing lateral position as a function of elastic number. **c** Heatmap illustrating the evolution of particle focusing downstream of the channel (indicated below) at varying PEO concentrations. In DI, DI + 22% glycerin, and 50 ppm PEO, particles focus near the inner half of the channel and progressively move toward the inner wall, illustrating the inertial flow regime. In the elasto-inertial flow regime, particles initially focus into three streams, then transition into two streams, and finally focus into a single stream. In the viscoelastic flow regime, at 5000 ppm PEO, particles rapidly evolve into a single stream

Viscoelastic migration and the equilibrium position of particles are strongly associated with medium elasticity^13,15^. The elastic number is independent of the flow rate and only depends on the fluid properties and device geometry. To investigate the effects of fluid elasticity, we evaluated particle migration in DI water with 5% NaCl (pure inertial flow, *El* = 0) and PEO solutions at 0–5000 ppm (*El* = 0–117) with 22% glycerol representing elasto-inertial and purely viscoelastic flows (*El* = 117). The PEO concentration was used to adjust elasticity rather than scaling device geometries to decouple these effects. The fluorescent streak velocimetry images and the corresponding brightfield images of particle distribution at the outlet of an R4 device at 100 µL/min in low (0 ppm PEO at *Re* = 5.7, *Wi* = 0, *De* = 0.6), medium (1000 ppm PEO at *Re* = 2.9, *Wi* = 13.2, *De* = 0.3), and high (5000 ppm PEO at *Re* = 0.36, *Wi* = 42.7, *De* = 0.04) elastic media are illustrated in Fig. 5a. The results show that at 0 ppm PEO (pure inertial flow, *El* = *0*), as expected, the particles focus off-center toward the inner wall. In the elasto-inertial flow at 1000 ppm PEO (*El* = 2.6), the focused stream is at the channel centerline. Increasing fluid elasticity to 5000 ppm PEO (purely viscoelastic flow, *El* = 117) shifts the focused stream toward the outer channel wall. Measurements of the lateral positions of particles as a function of *El* over a broader range in Fig. 5b confirm this observation. These measurements also suggest that elastic force begins to influence particle migration at approximately *El* > 1, with inertial effects mostly dissipating at *El* > 10. We then conclude that 1 <*El* < 50 is the range for the elasto-inertial flow.

The heatmap of the particle lateral position along the channel downstream in varying concentrations of PEO solution is shown in Fig. 5c. When *El* < 1 (for <50 ppm PEO solutions), the heatmap shows that particles progressively form two streams along the channel downstream. In this inertial fluid regime, the oppositely directed Dean drag *F*_*D*_ and inertial lift *F*_*L*_ forces equilibrate off-center, closer to the inner half of the channel^7^. When 1 <*El* < 50, particles initially focused into three streams transition to one stream. In this elasto-inertial fluid regime, the elastic force *F*_*E*_ balances inertial lift *F*_*L*_ and Dean drag *F*_*D*_, resulting in an equilibrium position at the channel centerline. When *El* > 50, particles rapidly migrate into a single stream (at 0.7 cm downstream) and gradually move toward the outer wall along the downstream length as *De* gradually increases. In this viscoelastic fluid regime, the elastic force *F*_*E*_ completely dominates in the vertical direction as the inertial force is negligible (*Re* = 0.36 and *El* = 117 for 5000 ppm PEO), leading to particle focusing in the vertical center plane where Dean drag *F*_*D*_ continuously drives the particles toward the outer wall.

### General focusing mechanics

**Fig. 6:**
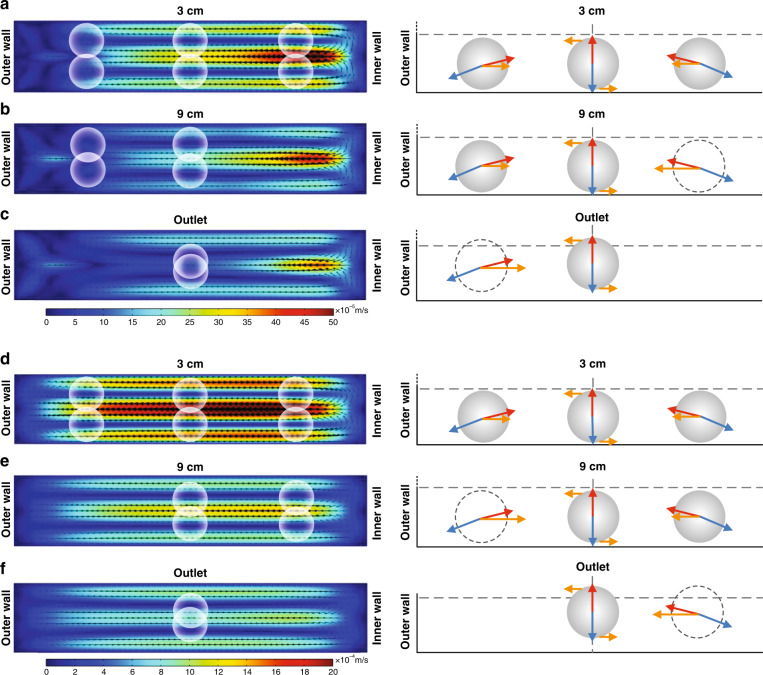
Evolution of particle migration in elasto-inertial flow in spiral microchannel. Numerical results of the velocity fields of the secondary flows due to Dean and elastic forces in the channel cross-section (left) and schematic (right) illustrating positions of focused particles and the force balance. The results are for the R2 channel, inside-out flow direction at 100 μL/min and (**a**) 3 cm, (**b**) 9 cm, and (**c**) outlet downstream positions. The results are also shown for 250 μL/min at (**d**) 3 cm, (**e**) 9 cm, and (**f**) outlet downstream positions. Blue, red, and orange arrows indicate inertial lift *F*_*L*_, elastic force *F*_*E*_, and drag force *F*_*D*_, respectively. Schematics show only the bottom half of the channel due to symmetry

Figure 6 illustrates numerical predictions of the velocity fields of secondary flows due to Dean and elastic forces in the spiral channel cross-sections, with the diagrams indicating the key forces involved in particle equilibration. As these numerical results show, the secondary flow in the elasto-inertial case is different from that in the inertial flow where two counter-rotating vortices are developed. The numerical results of the Dean flow evolution in Newtonian fluids, where the secondary vortices are symmetrically distributed along the channel height at the center of the channel at low *De* numbers, have been previously reported by us and others^40–42^. Here, vortices are modified due to the fluid elasticity (due to N_2_), leading to the lateral asymmetry of velocity magnitude in the secondary flow (Fig. S6). The secondary flow is stronger near the inner wall (for the inside-out flow direction). Note that the blockage ratio is relatively large in this work; thus, particles span both arms of the vortex, and it is the net force of the two opposing Dean drag forces that must be considered to determine the lateral position of the focused stream.

Particle focusing in spiral channels manifests in three major stages in the elasto-inertial flow. Randomly distributed particles rapidly form three distinct streams observed from the top of the channels (Stage 3 in Fig. 1). In this stage, the three streams persist, while the side streams gradually migrate toward the center. Here, the presence of the elastic force modifies the focusing patterns in the cross-section by balancing the shear-induced lift force in the vertical direction. This is illustrated in Fig. 6a. Next, in the transition stage (Stage T in Fig. 1), the side streams rapidly merge into the central stream, marking the beginning of the final stage where particles appear in a single stream. This evolution of the 3-stage particle focusing throughout the spiral channel is the result of the interaction of inertial, drag, and elastic forces. For the particles focused in the stream near the outer wall, the net drag force is toward the channel center. This net force is responsible for the toward-center migration of the outer streams observed in the top view (Fig. 1). According to the numerical model, Dean flow dominates at higher flow rates (e.g., 250 µL/min), while the modification of the secondary flow due to fluid elasticity is significant at low flow rates (e.g., 100 µL/min). Such a change in the secondary flow alters the relative intensity of the *F*_*D*_ near the channel center and near the channel bottom/top. Due to the high blockage ratio, particles near the channel side walls experience differing degrees of drag, leading to different disappearance sequences of the side streams observed in the transition stage between the low and high flow rate cases (Fig. 6b/e). The high blockage ratio of the 25 µm beads in our 50 µm high channel (*β* = 0.3 since *D*_*h*_ = 83.3 μm) means that beads span both the top and bottom portion of each of the Dean vortices, as illustrated in the numerical results of Fig. 6. The smaller 15 µm beads, which occupy 60% of the channel half height and have a blockage ratio of *β* = 0.18, still follow the same pattern of focusing dynamics (Fig. S5). The smaller 7.32 µm beads, which span only 29% of the channel half height and have a blockage ratio of *β* = 0.088, no longer follow the described pattern.

The numerical results predict that multiple pairs of Dean vortices appear at a low flow rate (100 μL/min) near the outer wall of the channel but diminish at a high flow rate (250 μL/min). The simulated sequential development of the secondary Dean flow suggests that the velocity profiles with asymmetric magnitude modify the force balance on particles near the wall at low flow rates. These results are consistent with the experimental results shown in Fig. 1a and Fig. 2a. For example, the stream near the outer wall disappears before the disappearance of the stream near the inner wall when the flow rate is ≤200 µL/min, while the opposite is observed at >250 µL/min (Fig. 1a).

While the net drag force causes the toward-center migration of the side streams in Stage 3, the change in the vertical particle positions leads to the final stage (Stage 1 in Fig. 1). As the particles in the side stream move closer to the center laterally, they simultaneously migrate in the vertical direction (Fig. 3) due to the change in the net force of the elastic force and shear-induced lift force in the vertical direction. Due to the modified velocity profile in the spiral channel, both the lift force and elastic force are not uniform in the cross-section (Figs. S7 and S8). This vertical movement is responsible for the rapid merging of the 3 streams into a single stream in Stage T. Particles near the top or bottom wall experience a stronger elastic force (Fig. S6), which alters the vertical position of the particles. Due to the symmetry of the Dean vortices in the *z*-axis, particles focus into two equilibrium positions along the channel height. This occurs in both Newtonian and viscoelastic fluids, with the key difference that the equilibrium position is near the channel center for the latter but is closer to the inner curvature for the former. The change in the vertical position alters the net drag force in the lateral direction, which causes a change in the migration direction of the center stream before and after the transition, which is especially evident at flow rates of 100–250 μL/min in Fig. 1a. In the position near the center, the net drag force due to the secondary flow can be zero (Fig. 6). As a result, the balance of the elastic force and inertial force (Fig. S6) is responsible for the formation of the three streams (six positions), and the net drag force due to the secondary flow brings the side stream to the center, resulting in two final equilibrium positions. Consequently, the lateral position of the final focused streams is not permeant, depending on the evolution of the secondary flow, as evidenced in the fluorescent images near the end of the channel (Fig. 1a).

The complex interactions between the inertial, elastic, and net Dean forces may also result in similarities in the focusing dynamics for the different flow directions in spiral channels, which is worth further discussion considering the streamwise asymmetry in spiral channels. As shown in Figs. 2–3, the general focusing dynamics remain the same for the outside-in and inside-out flow configurations for the spiral channels, flow, and rheological conditions explored herein. However, we may expect differences in the focusing dynamics in other cases. Let us assume an extremely simplified case, where the rate of change of Dean flow in the downstream direction C (C = d(De)/dx, where x is the downstream length) only affects Dean force within the normal range. In the case of C < 0 (inside-out flow direction), side positions in Stage 1 will move toward the center positions; however, due to the fast-attenuating net Dean force (F_D_ ~ De^1.63^)^48^, the side positions may never reach the center positions. Thus, there can remain 3 streams or 6 positions. In the case of C > 0 (outside-in flow direction), due to the fast-increasing net Dean force, the side positions in Stage 1 will rapidly merge into the center positions, leading to 2 final positions. In the case of C = 0, as the net Dean force does not change with time, we expect the same focusing dynamics for the two flow directions. In our case, C ≠ 0 but is not significantly large; thus, we observe similar focusing dynamics for the two flow directions, with the transition stage occurring at different time points depending on the flow rate and the radius of curvature. Nevertheless, significant differences can occur if the spiral is very small or the flow rate is high.

**Comparison and practical implications**

A number of publications have recently reported on viscoelastic migration dynamics in spiral channels. Table 2 summarizes the key nondimensional flow parameters (*Re, De, Wi*, and *El*) in these studies. The most notable shortcoming of these studies is the narrow range of the nondimensional parameters examined. Additionally, these studies examined 3D focusing behavior (positions) rather than focusing dynamics (downstream evolution). Xiang et al.^26^ proposed six stages of migration based on observations made at the end of the spiral channel at a single elasticity number (*El* = 4.3). The effect of the PEO concentration was not examined.

**Table 2:** Key nondimensional flow parameters in recent studies

Studies	Reynolds Number (*Re*)	Dean Number (*De*)	Weissenberg Number (*Wi*)	Elastic Number (*El*)
Xiang et al.^14^	0.031~1.007	0.006~0.0185	0.16~2.32	4.3~58.8
Xiang et al.^26^	0.04~9.66	0.006~1.407	0.17~41.8	4.33
Lee et al.^28^	0.007~1.5*	1~25	0.1~30	na
Kumar et al.^29^	20~67	na	1.3~5.3	0.03~0.08
This study	0.36~13	0.04~2.4	0~42.7	0~117

^*^Calculated from the reported flow rates and channel cross-sectional dimensions

Herein, we experimentally and numerically explored the evolution of viscoelastic focusing with respect to the channel length. We significantly expanded the range of El from 0 to 117, Re from 0.36 to 13, De from 0.04 to 2.4, and Wi from 0 to 42.7 and examined the change in the focusing patterns with respect to varying El. In addition, our computational results suggest that the second normal stress difference (N_2_) plays an important role during the transition of the focusing streams from three to one.

The concepts discussed in this work are based on rigid particles, while real-world applications often involve deformable cells. In the case of viscoelastic focusing in spiral channels, the deformability-induced force aligns with the elastic force and thus could potentially strengthen the toward-center net force. Therefore, the vertical focusing positions may move up slightly, but the final focusing patterns should not differ. It must be noted that the present work is based on dilute suspensions. As shown in our recent work^53^, different force interactions come into play in dense cell suspensions, such as whole blood, where strong cell‒cell interactions prevent cell focusing in microfluidic devices. That is the reason that whole blood generally must be diluted before processing in microfluidic devices. We do not anticipate whole blood to work directly in the viscoelastic spiral channels.

## Conclusions

In this work, for the first time, we experimentally demonstrated the evolution of particle focusing behavior along a channel downstream length at a high blockage ratio. We discovered that the vertical movement of the particles closely coordinated with their lateral migration, leading to slightly different focusing behavior depending on the flow rate and flow direction. We found that flow rate, device curvature, minimum *De*, and medium viscosity play important roles in particle lateral migration. Our results illustrate the full focusing pattern along the downstream channel length. Additionally, the simulation results sequentially predict that secondary flow dynamics in the channel cross-section are due to the combined effects of the Dean force and N_2_-induced secondary vortices along the channel downstream. Analyses of the particle lateral and vertical migration evolution demonstrated the underlying hydraulic force balance.

While the migration dynamics in straight channels have been explored, this work fills the knowledge gap in spiral channels, which exhibit more complex force interactions and unclear focusing dynamics. Our work delineates the migration and focusing patterns in carrier fluids with a wide range of properties. Thus, when developing spiral viscoelastic microfluidic devices, we may consider using only Stage 1 for rapid focusing and enrichment, or we may use 3D focusing in Stage 3 for spatial manipulation and cytometry applications. We may also consider adjusting the fluid properties (e.g., PEO concentration) when the pressure drop inside the device is a constraint.

## Supplementary information

Supplemental Information
