# Supplementary material for: Evolution of focused streams for viscoelastic flow in spiral microchannels
Source: Microsyst Nanoeng. 2023 Jun 6;9:73. doi: 10.1038/s41378-023-00520-4 (PMC10241945; doi:10.1038/s41378-023-00520-4)
Supplement: Supplementary file 1 — Supplemental Information [file 41378_2023_520_MOESM1_ESM.docx]

**Supplementary Information**

**Evolution of Focused Streams for Viscoelastic Flow in Spiral Microchannels**

Hua Gao, Jian Zhou,* Mohammad Moein Naderi, Zhangli Peng, and Ian Papautsky*

Department of Biomedical Engineering, University of Illinois Chicago, Chicago, IL 60607, USA

*Address correspondence to:

Dr. Jian Zhou ([jzhou88@uic.edu](mailto:jzhou88@uic.edu))

Dr. Ian Papautsky ([papauts@uic.edu](mailto:papauts@uic.edu))

Department of Biomedical Engineering

851 S. Morgan Street, 218 SEO

University of Illinois Chicago

Chicago, IL 60607, USA

**
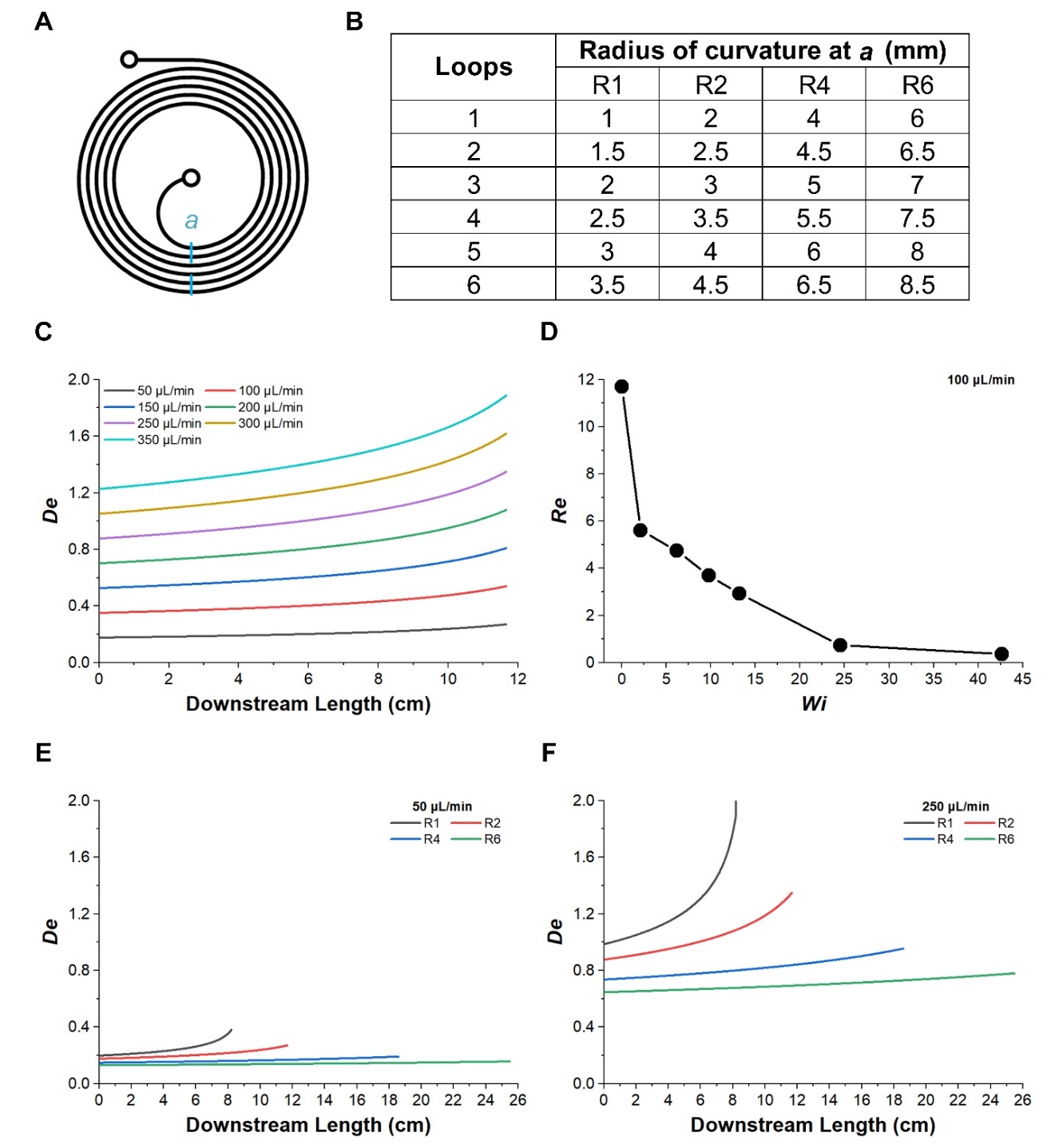
**

**Fig. S1.** **Details of device design.** (A) Schematic layout of spiral channel. (B) Table summarizing details of the four devices of different initial radii of curvature. Loop radius is given at position *a* for each device.

**
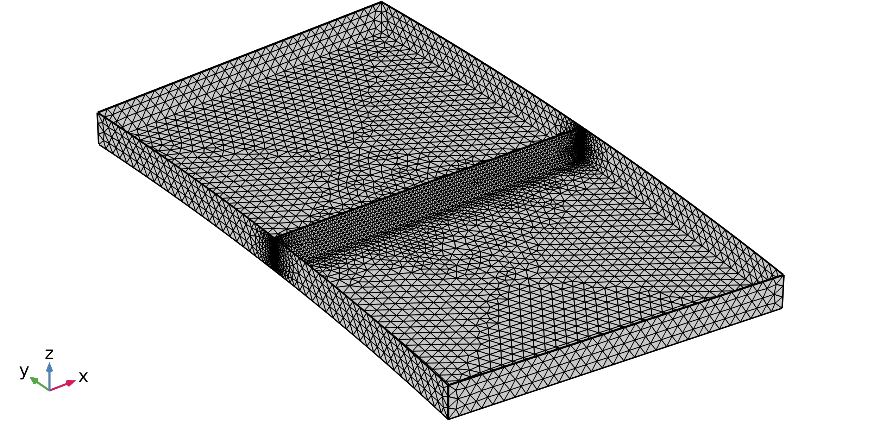
**

**Fig. S2. Model mesh configuration.** Finer mesh was used in the cross-section of interest.

**
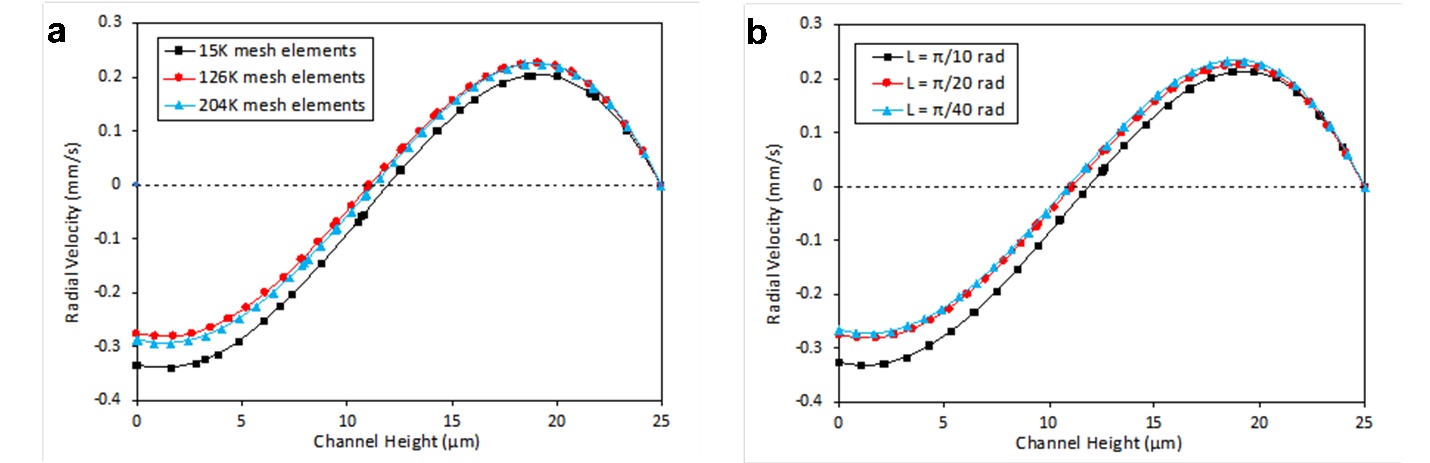
**

**Fig. S3. Dependency of simulation results on the number of mesh elements and the spiral segment length by plotting radial velocity along half the channel height.** (A) three different mesh configurations with 15000, 126000, and 204000 number of elements were evaluated, and the mesh-independency was observed beyond 126K elements. (B) Segment lengths of π/10, π/20 nd π/40 were simulated; channel length of π/20 ensured independence of the results from the domain length and provided longest possible channel segment for the flow to develop.

**
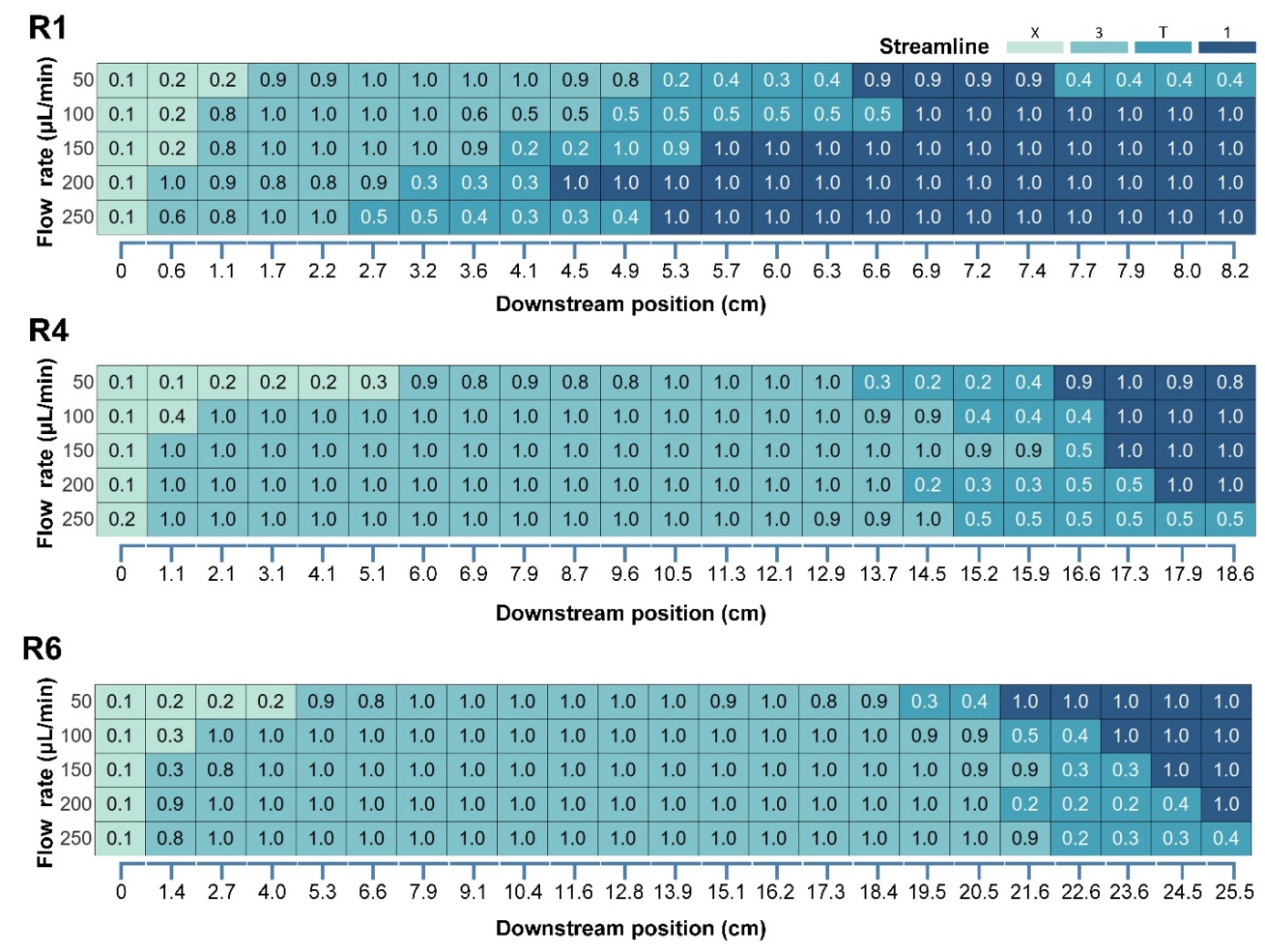
Fig. S4. Particle migration and focusing in R1, R2, R4, and R6.** Heat map illustrates the evolution of particle focusing streams. Each cell reports focusing quality (FQ = a/FWHM) of fluorescent streams. The color of each cell indicates the unfocused (X), three stream (3), transition (T) or single stream (1) regions.





**Fig. S5. Downstream evolution of focusing for 7.32 μm, 15 μm, and 25 μm diameter beads.** Stacked fluorescent streak images illustrating downstream evolution of particle focusing in R2 spiral channel (aspect ratio AR = 0.2) at 250 μL/min flow rate in 500 ppm PEO solution. The +/-125 scale indicates the normalized width of the 250 μm wide channel. Downstream position is indicated at the bottom of the panel set.


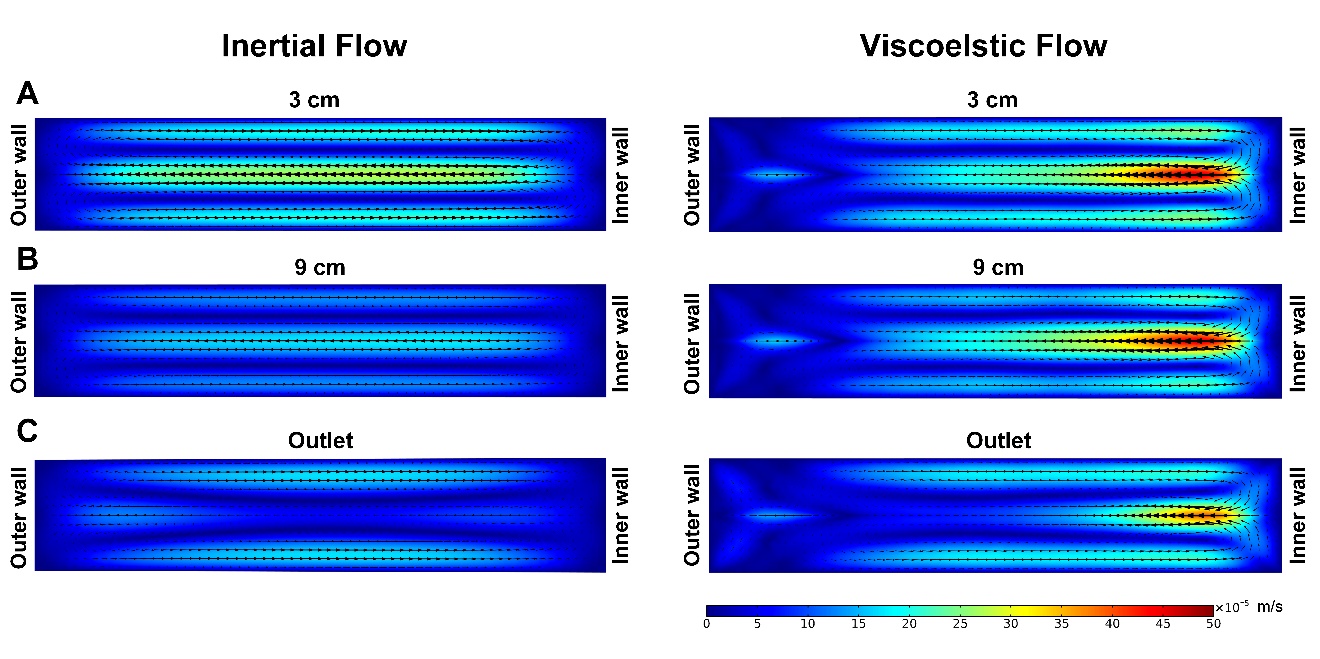


**Fig. S6. Comparison of inertial and viscoelastic flows.** Numerical predictions of the secondary Dean flow velocity profiles in the channel cross-sections show strong asymmetry in the viscoelastic flow. Results are for R2 spiral channel (aspect ratio AR = 0.2) at 100 μL/min flow rate in water (inertial flow) and 500 ppm PEO solution (viscoelastic flow). Cross-sections are shown at downstream positions of (A) 3 cm, (B) 9 cm, and (C) outlet.


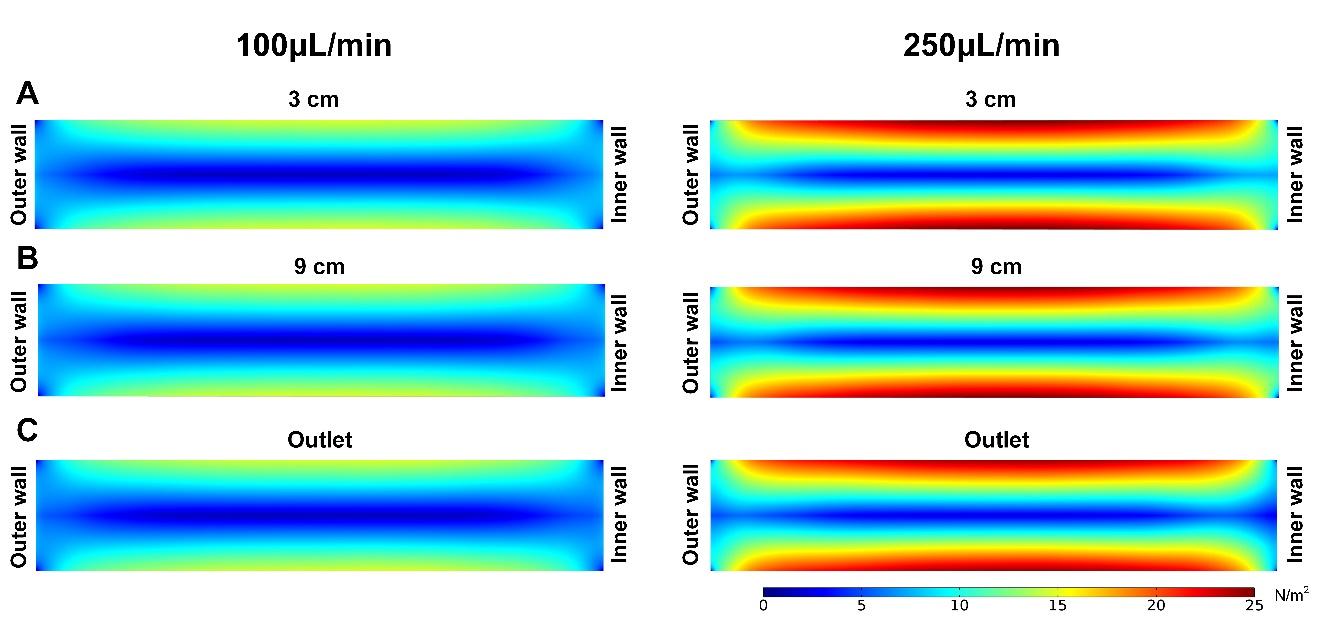


**Fig. S7. Numerical predictions of the first normal stress difference in the channel cross-sections in the downstream.** Results are for R2 spiral channel (aspect ratio AR = 0.2) at 100 μL/min (left column) and 250 μL/min (right column) flow rates at (A) 3cm, (B) 9cm, and (C) outlet (11cm) downstream. Dimensionless numbers at both flowrates are: *El* = 2.6, *Re* = 3.7/9.4, and *Wi* = 9.8/24.5.


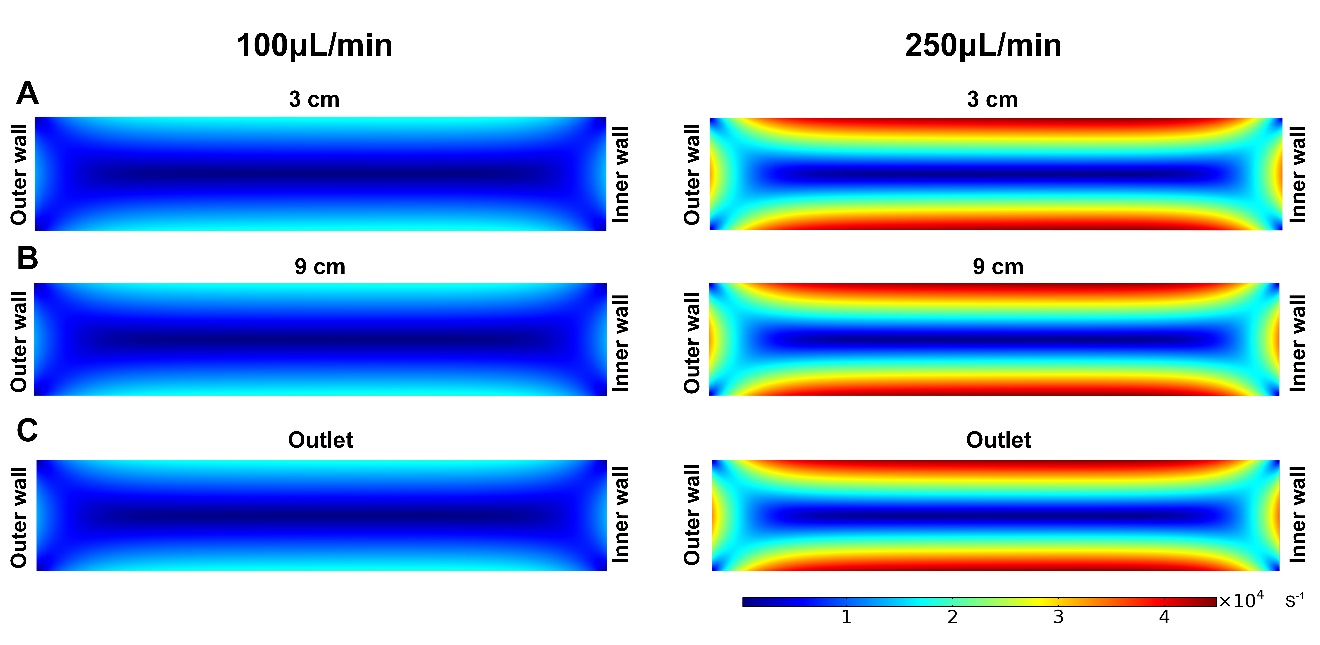


**Fig. S8. Numerical predictions of the shear rate in the channel cross-sections in the downstream.** Results are for R2 spiral channel (aspect ratio AR = 0.2) at 100 μL/min (left column) and 250 μL/min (right column) flow rates at (A) 3cm, (B) 9cm, and (C) outlet (11cm) downstream. Dimensionless numbers at both flowrates are: *El* = 2.6, *Re* = 3.7/9.4, and *Wi* = 9.8/24.5.
